# Characterising the impact of shift work on diet and glucose variability in healthcare employees living with type 2 diabetes: The Shift‐Diabetes study

**DOI:** 10.1111/dme.70262

**Published:** 2026-02-24

**Authors:** Rachel Gibson, Maria D'Annibale, Luigi Palla, Felicia Tejo, Barbara McGowan, Celine Vetter, Fabiana Lorencatto, Nick Oliver, Nicola Guess

**Affiliations:** ^1^ Department of Nutritional Sciences King's College London London UK; ^2^ Department of Public Health and Infectious Diseases University of Rome La Sapienza Rome Italy; ^3^ Department of Medical Statistics London School of Hygiene and Tropical Medicine and Medicine London UK; ^4^ Department of Diabetes and Endocrinology Guy's and St Thomas' NHS Foundation Trust London UK; ^5^ Department of Integrative Physiology University of Colorado Boulder Colorado USA; ^6^ Centre for Behaviour Change University College London London UK; ^7^ Department of Metabolism, Digestion and Reproduction Imperial College London London UK; ^8^ Nuffield Department of Primary Care Health Sciences University of Oxford Oxford UK

**Keywords:** continuous glucose monitoring healthcare employees, diet, shift work, type 2 diabetes, workplace health

## Abstract

**Aims:**

To characterise differences in dietary intake, glucose variability, and activity in free‐living healthcare shift workers with type 2 diabetes (T2D) across varying work conditions.

**Methods:**

Healthcare shift workers with T2D were monitored over 10 days, covering night shifts, day shifts, and rest days. Data were collected using blinded continuous glucose monitoring, activity trackers, and diet/sleep diaries. Within‐person comparisons were made for mean glucose (MG), coefficient of variation (CV), mean absolute glucose change (MAG), mean amplitude of glycaemic excursion (MAGE), continuous overlapping net glycaemic action (CONGA), dietary intake (food choices, nutrient intake), and activity/rest periods.

**Results:**

The study sample (*n* = 37; 89.2% women) were mainly employed as nurses or midwives (62.2%). Energy intake was highest (2199 kcal SD 648) on a day when a night shift was worked. Percentage of energy intake from sweet snacks was higher on a night shift compared with a rest day after a night shift (13.4 SD 12.0% vs. 7.8 SD 11.8%, *p* = 0.013). Night shifts had the highest eating occasions (7.0 SD 2.2) and rest after night (RAN) the lowest (3.4 SD 1.6), *p* < 0.001. No differences were reported for MG, MAGE, or CV. MAG and CONGA were higher for night shift compared with RAN shift (*p* = 0.029). Step counts were higher on night shift days (13,775, SD 4270 *p* = 0.016), and participants were awake longer (22.2 h SD 2.4 h, *p* < 0.001) compared with other day types.

**Conclusions:**

Night shifts are associated with prolonged wakefulness, increased activity, and distinct dietary behaviours. Tailored interventions are needed to support night shift workers with T2D in managing their condition effectively.

**What's new?**

**What is already known?**

- Night workers with type 2 diabetes are more likely to have higher HbA1c compared with non‐night shift workers.

**What this study has found?**

- Shift workers consumed more energy during a day where a night shift was worked.
- Small differences were observed for glucose variability, especially when a night shift was worked, likely driven by dietary differences.
- High activity levels were observed on workdays (>10,000 steps), with over half of steps registering during working hours.

**What are the implications of the study?**

- People with type 2 diabetes working mixed shift schedules with night work face particular challenges in managing their condition.

## 1 INTRODUCTION

There is convincing evidence for an association between shift work exposure (e.g., working outside of standard work hours of between 07:00 and 19:00) and poorer cardiometabolic health outcomes.^1^ Large‐scale prospective studies in nurses suggest a dose–response increase in type 2 diabetes (T2D) risk with years of rotational shift work.^2^ Disruption of the body's circadian rhythms are likely to contribute to the detrimental effects of shift work on health through physiological changes in the endocrine system coupled with modifiable health behaviours.^3^

While observational studies and systematic reviews show an association between shift work exposure and T2D risk^2^, ^4^ there are limited studies investigating the impact of shift work in employees diagnosed with T2D. A small study conducted in Thailand observed that hospital night workers with T2D experience higher glycated haemoglobin (HbA1c) compared with day workers.^5^ Limited research has investigated the changes in blood glucose over a mixed shift work and rest schedule. An observational study in shift and non‐shift‐working employees without T2D reported elevated nighttime mean blood glucose (MBG) and higher 03:00 blood glucose in night workers^6^; however, a key limitation was the absence of dietary intake recording.

As dietary modification is one of the key first line intervention strategies in the management of T2D,^7^ understanding how dietary intake changes between different shift types is important. There are numerous studies showing differences in dietary behaviours in shift workers compared with non‐shift workers, for example, shift workers are more likely to report increased eating frequency, fewer healthy food choices^8^ and a redistribution of dietary energy to the night.^9^ A recent systematic review identified limited studies investigating how food choices change within individuals between different types of shifts^8^ and none were reported to have been conducted amongst shift workers living with T2D.

Understanding the impact of shift work on T2D is important given the higher prevalence of T2D in UK shift workers compared with day workers^10^ and the health outcomes associated with elevated glucose control in people with diabetes.^11^ From an economic perspective, this can lead to early exit from employment, increased sickness work absence,^12^ and increased health care costs.^13^

## 2 AIMS

Shift‐Diabetes is a mixed methods case study designed to address current research gaps which limit our ability to develop lifestyle interventions tailored to the needs of night shift workers with T2D.^14^ The qualitative findings have previously been published.^15^, ^16^ This article reports the results from the quantitative study, the aim of which was to characterise differences in glucose variability, dietary intake, and activity between night shifts, day shifts, and days off in healthcare sector workers with T2D.

## 3 METHODS

### 3.1 Study design and ethical approval

The Shift‐Diabetes study is an observational case study and was approved by King's College London BDM Research Ethics Subcommittee (HR‐19/20‐14630) and conducted in accordance with the Declaration of Helsinki. The Shift‐Diabetes study is registered at ISTCTN (Ref: 11764942). Informed consent was gained before research activities were conducted.

### 3.2 Participants and recruitment

Employees aged 18–60 years, with T2D who worked shifts in a hospital or residential care setting were recruited across the UK through (i) posters placed on staff notice boards and newsletters in hospitals and care homes, (ii) targeted social media posts (Instagram, Facebook, Linked In and Twitter), (iii) Nursing Standard on‐line website, and (iv) Diabetes UK website ‘take part in research’. All interested participants self‐referred directly to the Shift‐Diabetes study team. Unless participants self‐disclosed taking part in the study employers were not made aware that staff had participated. Participants were eligible if they reported working a mixed shift schedule including night shifts and were managing their condition with lifestyle and/or medications not associated with high hypoglycaemic risk (e.g., acarbose, metformin, SGLT2 inhibitors, DPP‐4 inhibitors). In line with the UK definition, night work was defined as ‘a period of at least 3 h of works between 23:00 and 06:00’.^17^ Eligible participants needed to work four or more night shifts in a typical month. We did not set a minimum requirement for duration of employment in night work or time since diagnosis of T2D. Interested participants were sent an information sheet and completed a prescreening questionnaire and a screening phone call with the Research Assistant. Those who met the inclusion criteria were invited to either join the study in person, at the Metabolic Research Unit at King's College London or remotely, via videocall where the study was explained, and consent was obtained. Recruitment to this study took place from September 2020 to February 2023. Participants were compensated £60 for taking part in the study. All participants received a report detailing their dietary intake, activity, sleep, and blood glucose after completing the study.

### 3.3 Procedure

The protocol for the Shift‐Diabetes study has been previously published.^14^ This study was significantly impacted by COVID‐19, and a remote study option was developed and approved to mitigate impact of suspension of face‐to face research. Dates for the monitoring period were agreed in advance with participants as the period needed to cover 10 days including a minimum of three‐night shifts (arranged in either three consecutive night shifts or two consecutive night shifts and one single night shift), a minimum of one other work shift (any other type of shift not classified as ‘night shift’) and days off. Participants were asked to stop taking vitamin C supplements 1 week before and during the monitoring period due to potential for interference with the continuous glucose monitor (CGM) accuracy. Participants were required to attend two study visits either in person at the Metabolic Research Facility at King's College London, Waterloo Campus or virtually via an on‐line video platform (e.g., MS Teams) with the Research Assistant.

During the first visit, body weight measurement was taken by the Research Assistant for participants attending in person, or for remote study visits, participants were asked to self‐report weight. Body mass index (BMI, kg/m^2^) was computed for each participant. Participants were asked to complete a health and lifestyle questionnaire that recorded information on (i) occupational variables (job role, years of shift work and duration of working hours), (ii) sleep, (iii) chronotype (self‐reported identification of being a ‘morning’ or ‘evening’ person) and (iv) relevant medical history (last HbA1c, if known, medications, nicotine product usage, physical activity, and co‐morbidities). Participants were given a blinded activity and sleep monitoring device (ActiGraph GT9X Link; ActiGraph LLC) to wear for 10 days on their non‐dominant wrist. If work restrictions required a bare below the elbow policy, participants could wear it on their waist during work hours. Participants were fitted with or guided to insert a blinded CGM (Dexcom G6, Dexcom). All participants were provided with a paper 10‐day diary to record all their food and drink, working hours, and sleep. Written and verbal instructions were provided about how to complete the diary. Printed images of portion sizes were provided to help with the estimation of foods.^18^ Participants were asked to record the time and location of each eating or drinking occasion. During the monitoring period, participants were asked to not change their usual diet or activity levels.

At the second study visit, following completion of the 10‐day monitoring period, the CGM and actigraphy devices were removed, and further questionnaires were completed to collect information on (i) general diet for the recording period (e.g., check list of types of milk and cooking methods to aid dietary analysis) and (ii) sleep quality information for the monitoring period PROMIS ‘Sleep Related Impairment’ and ‘Sleep Disturbance’ questionnaires.^19^ The Research Assistant checked the diet record on receipt for completion and checked with the participant to clarify food items or eating times if unclear.

#### 3.3.1 Dietary data

Dietary intake was recorded in a 10‐day food record, adapted from a UK validated 7‐day food record,^20^ and was coded by Associate Registered Nutritionist (MD) using Nutritics nutritional computer software (v5.09, Dublin, Nutritics, 2019). Incomplete days of recording (as agreed with participant at diet record check) were excluded from analyses. Nutritional intakes (energy, macro nutrients, fibre, sodium, and caffeine) were derived from UK Nutritional Database and manufacturer declarations when exact matches were not available. Selected foods and beverages were categorised into sugar‐sweetened beverages (SSBs), whole grain foods (based on the British Dietetic Association classification^21^), fruit and vegetables (excluding juices and smoothies), and discretionary high sugar foods (based on Food Standards Scotland definition^22^). Eating windows were defined as duration (hours:minutes) between first and last energy intake (eating/beverage >0 kcal).^23^ ‘Eating’ occasions were classified as any energy intake (>0 kcal) separated by >15 min preceding or succeeding.^24^

#### 3.3.2 Continuous glucose monitoring

A blinded CGM device took glucose readings in the interstitial fluid every 5 min during the 10 days of the monitoring study. The CGM data were imported into the Dexcom Clarity platform (Dexcom, Dexcom Clarity, https://clarity.dexcom.eu/) and then a .csv file exported for analysis. Validated metrics of glycaemic variability were calculated using open access software (Easy‐GV, Version 10).^25^ For missing data ‘Interpolate Missing’ function was used. Off‐scale low values were replaced with the CGM system's lowest reportable value (2.2 mmol/L, 40 mg/dL). A priori primary outcome variables were: coefficient of variation (CV), mean amplitude of glycaemic excursion (MAGE), percentage of time above 180 mg/dL (10 mmol/L), above very high (>250 mg/dL/>13.9 mmol/L). Additional outcomes reported were MBG, mean absolute glucose change (MAG), continuous overlapping net glycaemic action (CONGA), and glucose management indicator—estimated HbA1c.

#### 3.3.3 Sleep and physical activity data

Sleep and physical activity were measured using the combined actigraphy and accelerometer device (ActiGraph GT9X Link; ActiGraph LLC) and by self‐report sleep and physical activity in daily record. Data were collected over the 10‐day monitoring period in 30‐s epochs. Physical activity was defined by intensity and duration—time in light, moderate, vigorous, and very vigorous—and the total number of steps. Sleep activity was recorded by the actigraphy device and processed by ActiLife software (ActiLife V6, ActiGraph LLC). First, sleep periods were automatically detected using the ‘custom sleep scoring algorithm’, for analysis on 30‐s epochs data. Sleep times were checked for accuracy with the data recorded in the sleep logs. Where there were disagreements between the sleep log and activity monitor, the participant was contacted to verify; when this was not possible (and both data sources were available) the data from the activity monitor was used to estimate sleep. From these data, the variables extracted were sleep duration, sleep offset and onset, sleep efficiency, and sleep latency.

#### 3.3.4 Working hours and classification of day types

**FIGURE 1:**
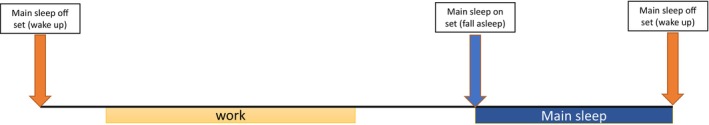
Example of ‘*behaviour determined day*’ measurement period used to determine the unit of comparison.

Participants recorded the start and end of each shift in the diet record; this was then checked with the Research Assistant at the second study visit. Where participants reported part shifts (<5 h), data for these days were not included in the analysis (as this equated to less than half a typical shift duration). The classification of ‘day type’ was determined as the ‘behaviour determined day’ applying a previous protocol developed for shift work dietary analyses, where a ‘day’ was defined as the period between two main sleep periods, that is the sleep period of longest duration in each 24‐h calendar day.^26^ Each day type began with the sleep offset (wake up from main sleep), prior to the shift worked/rest, until the following sleep offset (wake up time of the following main sleep) (Figure 1). Therefore, each ‘day’ would be of variable duration. Different day types were differentiated based on the shift worked or rest days. Therefore, 4‐day types were detected: non‐night shift, night shift, day off, and rest after night (RAN) shift (e.g., a night shift ends, and another shift is not scheduled within the next 24 h). Worked example Figure S1.

The aim was to compare whether dietary and glycaemic variables of interest were significantly different across the 4‐day types identified.

### 3.4 Statistics

An a priori sample size was estimated prior to study commencement. The estimated sample size required was 70 based on a power of 80% (5% type I error) of a two‐sided *t*‐test to detect a shift type difference in the CV of 10% from 32% (average CV reported previously in people living with T2D^27^) to 42% when measuring the CV based on repeated measures every 5 min in independent subjects. Statistical analyses were conducted using SPSS‐29 (IBM SPSS Inc.). Baseline characteristics were summarised using frequencies (categorical variables); mean and standard deviation (SD) for continuous variables. The distributions of continuous variables were explored; normality was not verified therefore Wilcoxon Signed‐Rank and Friedman non‐parametric tests were used to test within‐person differences across day types. To account for differences in ‘day’ duration and shift duration energy adjusted intakes are reported as percentage of energy intake or grams per 1000 kcal. For significant test results (*p* < 0.05), post hoc tests were performed to test for differences between groups. Adjusted *p*‐values (Bonferroni correction for multiple comparisons) are reported. Incomplete data recording (i.e., where data were only available for part of a day) from the CGM or diet record were excluded prior to conducting analyses.

## 4 RESULTS

### 4.1 Enrolment and data collection

A total of 84 participants were screened for eligibility (of which 27 were ineligible), 47 consented to take part, and 39 completed the study Figure S2, with the COVID‐19 pandemic significantly disrupting recruitment. Online visits were completed for 28 (75.7%) participants. Due to the ad hoc nature and short notice changes to work rotas, not all participants recorded all day types during the monitoring period. One participant did not have a shift schedule that met protocol requirements (a non‐night shift included in their monitoring periods), and their data were excluded from analyses. Additionally, CGM recording failed for one participant, and one participant had inaccurate work hour records to match to sleep and activity data. The final sample size for the dietary analyses was 37, with 36 for the glucose analyses. There were 32 participants with complete activity data.

### 4.2 Study sample characteristics

Most participants worked in England (97.3%) with the remaining working in Scotland. The study sample were predominantly women (89.2%), white (62.2%), worked as a Registered Nurse or Midwife (62.2%), and in a hospital setting (89.2%) (Table 1). The mean age of participants was 48 years (SD 7.2 years). Most reported working an irregular shift pattern (67.6%). The mean reported total years of shift work 15.6 (SD 11.0 years) and working a mean of 7.2 (SD 2.5) night shifts per month. Mean years since T2D diagnosis was 5.4 (SD 4.1 years) and most participants reported taking medication for diabetes (83.8%).

**TABLE 1:** Baseline characteristics of participants taking part in the Shift‐Diabetes study (*n* = 37).

	*n*	%
Women	33	89.2
Country		
England	36	97.3
Scotland	1	2.7
Ethnicity		
White	23	62.2
Black/African/Caribbean /Black British	8	21.6
Asian/Asian British	6	16.2
Job role		
Registered nurses and midwives	23	62.2
Nursing or healthcare assistants	8	21.6
Other	6	16.2
Workplace		
Hospital	33	89.2
Residential care	4	10.8
Shift pattern		
Irregular	25	67.6
Rotating	10	27.0
Other	2	5.4
Predominantly desk‐based job role	4	10.8
Current cigarette smoker/vape user	6	16.2
Non‐drinker	8	21.6
Any physical activity last 7 days	32	86.5
Take diabetes prescribed medication	31	83.8
Attended Diabetes education programme	30	81.1
Diagnosed high blood pressure	15	40.5
Diagnosed high cholesterol	14	37.8
Living with overweight	7	18.9
Living with obesity	27	73.0

	Mean	SD
Mean age, years (SD)	48.2	7.2
Mean working years that have included night shift work (SD)	15.6	11.0
Mean typical duration of weekly working hours (SD)	37.5	4.2
Mean typical number of night shifts worked per month (SD)	7.2	2.5
Mean body mass index, kg/m^2^ ^a^	33.8	5.8
Mean self‐reported latest HbA1c measurement, mmol/mol (%)^b^	55 (7.2)	10 (3.1)
Mean years since diabetes diagnosis	5.4	4.1

^a^ BMI self‐report *n* = 28 (remote study visits).

^b^ HbA1c self‐reported *n* = 20 (17 participants did not provide information regarding last HbA1c measurement).

### 4.3 Working hours and rest days

In total, 354 days were captured across 37 participants. Over the 10‐day monitoring period, three or more night shifts were captured in 31 (84%) participants. Mean shift durations were 11.4 h (SD 1.8) for day shifts and 12.2 h (SD 0.63) for night shifts (*p* = 0.005) (Table 2). All night shifts met the criteria of three or more hours worked between 23:00 and 06:00 (mean start 19:45 h range 18:00–21:15, mean end 08:07 h range 06:00–08:30). At least 2 day shifts were captured in 28 (76%) participants. Two or more ‘days off’ were recorded in 30 (81%) of participants. A RAN shift was not captured for one participant. One participant recorded part shifts (<5 h duration).

**TABLE 2:** Comparison of nutrient and food intake by behavioural determined day types mean (SD) *n* = 37.

	Rest after night		Day off		Night shift		Day shift		*p*
	Mean SD								
Day type duration, h	17.2	2.7	23.55	1.0	26.9	2.5	24.6	1.5	<0.001^a^
Shift duration, h	n/a		n/a		12.2	0.63	11.4	1.84	0.01
Time awake, h	9.6	2.8	15.8	1.3	22.2	2.4	17.1	1.2	<0.001^b^
Eating window, h	6.2	4.5	10.9	2.2	18.8	4.3	13.7	2.1	<0.001^c^
All eating occasions	3.4	1.6	5.2	1.5	7.0	2.2	5.3	1.7	<0.001^b^
Tot energy kcal	1284	625	1889	620	2199	648	1836	591	<0.001^d^
% EI during work hours	n/a	n/a	n/a	n/a	33.3	19.6	52.9	16.6	<0.001
Carbohydrates (% EI)	42.8	13.3	41.8	7.9	43.6	8.5	43.6	8.8	0.36
Tot fats (% EI)	36.0	13.3	39.2	8.0	39.0	8.2	37.1	8.1	0.17
Saturated fats (% EI)	11.3	6.0	13.5	5.0	13.2	3.9	13.1	3.8	0.038^e^
Protein (% EI)	19.6	8.1	17.8	4.3	17.2	3.6	18.8	5.0	0.33
Free sugars, g/1000 kcal	24.3	21.3	20.3	10.6	20.6	12.0	22.1	14.6	0.70
Salt, g/1000 kcal	3.1	1.7	2.9	0.9	3.3	1.3	3.1	1.1	0.52
Fibre, g/1000 kcal (AOAC)	11.4	7.5	11.2	5.1	10.3	3.9	11.1	5.2	0.77
Fruit, g/1000 kcal	28.2	59.0	46.2	60.0	59.6	56.5	69.1	71.5	<0.001^f^
Vegetables, g/1000 kcal	155.6	171.7	128.1	161.6	109.2	139.8	115.1	127.9	0.87
Wholegrains, g/1000 kcal	15.0	33.9	16.9	31.6	22.2	28.5	23.5	37.4	0.011^e^
SSB % EI	2.2	4.9	0.7	1.5	0.8	1.8	1.2	2.6	0.19
Sweet snacks % EI	7.8	11.8	12.1	10.9	13.4	12.0	11.6	12.9	0.016^g^
Alcohol, g/day (ethanol)	3.6	11.6	3.6	9.2	0.9	5.2	2.7	7.9	0.15
Caffeine, mg/day	54.6	72.0	85.2	98.8	164.8	175.5	104.2	106.2	<0.001^h^

*Note*: AOAC % EI percentage energy intake (total), SSB sugar‐sweetened beverages. One participant recorded not eating during their night shift (included in analyses). Comparisons between 4‐day types *n* = 36 (one participant did not record ‘rest after night’). Comparisons between shift (paired) = 37. Wilcoxon signed‐rank test compared pairwise difference between 2 day types. Freidman conducted between three or day types, with post hoc test applied if significant. Asterisks denote significant differences from post hoc tests, with adjusted *p*‐values.

^a^ Rest after night versus day off, rest after night versus day shift, day off versus night shift all *p* < 0.001.

^b^ All pairwise comparisons *p* < 0.001 except day off versus day shift which was not significant.

^c^ Rest after nights versus day shift, rest after night versus night shift, day off versus night shift all *p* < 0.001; Rest after night shift versus day off *p* = 0.028; day off versus day shift *p* = 0.016.

^d^ Rest after night versus day shift *p* = 0.003, rest after night versus day off *p* = 0.002; rest after night versus night shift *p* < 0.001.

^e^ No significant difference in post hoc analyses for adjusted *p* value.

^f^ Rest after night versus day shift *p* = 0.003; rest after night versus, ‘night shift’ *p* = 0.002.

^g^ Rest after night versus night *p* = 0.013.

^h^ Rest after night versus day off *p* = 0.032; rest after night versus day shift *p* = 0.010; rest after night versus night *p* < 0.001; day off versus night shift *p* = 0.010; day shift versus night shift *p* = 0.032.

### 4.4 Behavioural day characteristics

The duration of time awake was not significantly different between days when a night shift or day shift was worked. The behavioural day (sleep off set to sleep off set) was longest when a night shift was worked (day duration 26.9 h SD 2.5) this was significantly longer than rest day (*p* < 0.001) or RAN shift (*p* < 0.001). RAN shift was significantly shorter than all other day types (17.2 h SD 2.7). Two participants did not have a sleep period within 12 h of ending a night shift. The behavioural day was taken as an extended ‘night shift’ day with no RAN shift.

### 4.5 Dietary intake

#### 4.5.1 Overall summary dietary intake

Overall, 10.8% of participants met the recommendations for daily fibre intake (mean intake 20.1 g/day SD 10.3) and 5.4% for fruit and vegetables (mean fruit 98.8 g/day SD 78.3, vegetables 222.2.4 g/day SD 586.5). Four participants reported not eating any fruit during the monitoring period. Diet intakes across the monitoring period are detailed in Tables S1 and S2.

#### 4.5.2 Dietary intakes across behavioural day types

The highest difference in mean energy intake was reported on a day where a night shift (2199 kcal SD 648) compared with other days (RAN, 1284 kcal SD 625; day off 1889 kcal SD 620; day shift 1836 kcal SD 591) was worked (Table 2). A higher proportion of energy (kcal) was consumed during working hours on a day shift (52.9% SD 16.6) compared with night shift (33.3% SD 19.6, *p* < 0.001), even though the night shift had the longest duration of eating window (18.8 h SD 4.3) and number of eating occasions (7.0 SD 2.2). There was no difference in the percentage energy intake from carbohydrate, total fat, or protein across day types. Energy intake from sweet snacks was significantly higher on night shift days compared with RAN shift (13.4% SD 12.0 vs. 7.8% SD 11.8, adjusted *p* = 0.013).

### 4.6 Glucose variability

The mean percentage data collection over the 10‐day period was 94% SD 16.1%. No participant had <70% of data. Across all participants on average 68.0% of time during the monitoring period was spent within the range of 3.9–10 mmol/L (70–180 mg/dL) (Table S3). No differences were observed across day types for MBG, standard deviation, CV, MAGE or time in range (Table 3). Post hoc tests showed MAG and CONGA to be higher during a night shift compared with ‘RAN’ (adjusted *p* = 0.029, *p* = 0.016 respectively).

**TABLE 3:** Comparison of glucose variability metrics by behavioural day type mean (SD) (*n* = 34).

	Rest after night		Day off		Night shift		Day shift		*p*
	Mean SD								
Mean blood glucose (MBG), mmol/L	9.1	2.2	9.1	2.2	9.0	1.9	9.0	2.0	0.98
SD, mmol/L	1.7	0.7	1.7	0.6	1.9	0.6	1.7	0.5	0.13
Coefficient variation (CV), %	18.9	5.8	18.3	4.7	20.6	5.1	18.9	4.2	0.040*
Mean absolute glucose change (MAG), mmol/L	2.9	1.6	2.8	1.5	3.1	1.4	2.9	1.2	0.015**
Mean amplitude of glycaemic excursion (MAGE), mmol/L	4.7	2.1	4.6	1.7	4.9	1.7	4.6	1.4	0.46
Continuous overlapping net glycaemic action (CONGA), mmol/L	2.0	0.6	2.1	0.6	2.4	0.7	2.2	0.6	0.025***
Time in range (3.9–10.0 mmol/L), %	69.2	30.6	68.1	28.8	67.3	27	69.7	27.7	0.09
Time in severe (>13.9 mmol/L), %	7.3	16.0	7.2	15.6	7.4	13.2	5.8	12.9	0.32

*Note*: Exclusions: no CGM data (*n* = 1), no CGM recorded for night shift (*n* = 1), no ‘rest after night’ in monitoring period (*n* = 1). Freidman conducted and post hoc test applied if significant and adjusted *p*‐values reported.

* Not significant in post hoc tests.

** *p* = 0.029 ‘rest after night’ versus ‘night shift’.

*** *p* = 0.016 ‘rest after night’ versus ‘night shift’.

### 4.7 Activity

The number of steps was higher on a day when a night shift was worked (13,775, SD 4270) compared with all other day types (*p* < 0.05) (Table 4). There was no difference between steps during the shifts. Sedentary time was higher during the ‘RAN shift’ (70.0 SD 13.5% time) compared with when a night shift was worked (60.1 SD 11.5% time), *p* < 0.05. Less than 0.05% time in vigorous activity was recorded on RAN and day off (data not presented); no participant recorded activity in the very vigorous range. Most participants reported none/slight sleep impairment (62.2%) and the most frequently reported chronotype was ‘evening’ (37.8%); 54.1% were in the high sleep apnoea risk category. Self‐reported sleep parameters are shown in Table S4.

**TABLE 4:** Physical activity and sleep comparison across types of workdays and shifts (*n* = 35).^a^

Mean SD	Rest after night		Day off		Night shift		Day shift		*p*
Total steps, *n*	6106	4407	10,017	4343	13,775	4270	11,414	3172	<0.001*
Steps during shift, *n*	—	—	—	—	7506	2972	7753	2583	0.48
% steps at work	—	—	—	—	55.1	14.5	67.6	11.9	<0.001
Total time sedentary, %	70.0	13.5	64.1	10.7	60.1	11.5	63.9	11.7	<0.001**
Sedentary time work, %	—	—	—	—	55.1	18.2	50.4	19.4	0.60
Total time light, %	22.6	8.9	26.5	7.0	31.4	8.2	29.4	8.9	<0.001***
Light time at work, %	—	—	—	—	37.5	14.1	42.0	14.9	0.019
Total time moderate, %	7.4	6.9	9.3	5.4	8.5	5.7	6.0	5.3	0.006****
Moderate time at work, %	—	—	—	—	7.4	7.3	6.9	8.4	0.94

*Note*: Asterisks denote significant differences from post hoc tests.

^a^ One participant did not have a rest after night in the monitoring period; two participants did not capture day shift activity data. Wilcoxon signed rank test compared difference between two groups. Freidman conducted between three or more groups, with post hoc test applied if significant.

* Rest after night versus day off *p* = 0.012, night versus day shift *p* = 0.006, all other pairwise comparisons *p* < 0.001.

** Night shift versus rest after night *p* < 0.001.

*** Rest after night versus ‘day shift’ *p* = 0.003, rest after night versus night shift; *p* <0.001.

**** Day shift versus night shift *p* = 0.016, rest after night versus ‘night shift’ *p* = 0.40.

## 5 DISCUSSION

To date, no study has reported at the granular level glycaemia, dietary intake, activity and sleep across different working hours in employees with T2D, and these data are needed to develop lifestyle interventions tailored to the needs of night shift workers with T2D. This study observed that shift workers tend to consume less energy during a night shift, potentially leading to overconsumption of poor‐quality either side of working hours. This study also found that sleep and activity were variable across the different shift times. An important incidental observation was the variability in ‘day’ length depending on the time of shift worked. For our primary glucose variability measures. We also found that glycaemic variability was greatest during a night shift and was likely due to dietary intake.

The granular data collected enabled the examination of dietary intake over different shift patterns. Observations showed that energy intakes were higher during a day where a night shift was worked. In addition, most of the energy was not consumed during the night shift itself, and that the greatest proportion of energy from sugary snacks occurred during the night shift. These data, along with previous qualitative findings from this cohort, suggest that night shift workers are not eating much during the night, and that the food workers do eat comes from high sugar snacks. It seems that workers are then overcompensating either side of their night shift with high saturated fat, high energy foods—this extended ‘eating window’ reflects the longest behavioural day. These findings align with the qualitative research where the food environment was seen as an important barrier to making healthy food choices at work. During night work, emotions, such as stress, boredom, and tiredness were perceived as promoting less healthy food choices, as was not being able to resist sweet foods. These data provide a starting point for behaviour interventions targeted specifically at night shift workers with T2D.

There are limited studies exploring intraindividual differences across different types of shifts.^8^ A previous observational study in UK women shift‐working nurses found no difference in energy intake across type of shift worked with energy intake^9^; however, a different approach was used to define duration of energy intake. Suggesting how dietary intake is measured and reported is an important consideration when comparing study results. A previous study in rotating shift nursing staff in Israel reported within‐person, day to night shift intakes increased for energy, protein, and saturated fat, and the researchers noted that difference may relate to food environment.^28^ Previous studies, in non‐healthcare workers have found higher sugar intakes through SSB's—this was not observed in the current study, likely due to healthy beverage vending implementation across NHS sites.

This study also found that Shift‐Diabetes participants were in general less healthy than the UK adult population. Only 5% met fruit and vegetable intake guidelines, compared with 33% of the general UK adult population.^29^ Themes from the Shift‐Diabetes qualitative study reflect the overall dietary profile of low fruit, vegetables, and fibre and higher saturated fat and free sugars.^16^ Barriers reported included the ability to adjust eating patterns in relation to changing shifts, the ability to make healthier food choices because of working at night, with a reliance on convenience. Also, a perception of not being possible to eat healthy when you work night shifts. The current study did not find any differences in nutrient intakes, and the number of eating occasions between when a day shift was worked compared with a day off suggests similar eating behaviours between these day types. In summary, the observational findings of this research support the previous behaviour change recommendations from the Shift‐Diabetes qualitative findings—to consider interventions that target knowledge, decision making, environmental cues and social influences to improve dietary choices.^16^

The duration of eating windows has gained increasing interest in nutrition and health research, with time‐restricted interventions (reducing from 14 to 11 h) showing potential benefits in US firefighters when combined with a Mediterranean diet in those at elevated cardiometabolic risk.^30^ The current study found eating windows to be of variable length based on the type of shift worked. During days when night or day shifts were worked, the eating windows were over 14 h duration, a duration previously associated with overweight and obesity.^23^ The longer eating window is a likely characteristic of the UK healthcare sector where standard shifts are typically 12 h. The longer eating window when a night shift is worked was also found in a previous study in Brazilian miners, with night work associated with a longer eating window.^31^ Although observational studies have reported that irregular daily energy intake is associated with poorer cardiometabolic health outcomes in the general population,^32^ less is known about the fluctuation of eating windows and the potential impact on health in people living with T2D. The fluctuating ‘day length’ across different working days is a novel finding and warrants further investigation. This observation also reflects findings from the Shift‐Diabetes qualitative study where participants reported challenges of adapting eating times across a changing shift schedule.^16^ Experimental studies would be valuable to identify whether specified time of eating approaches may be an effective strategy to manage cardiometabolic risk in night shift workers.

While average glucose concentrations have been found to be elevated in night shift workers compared with non‐night shift workers,^5^ the relative contribution of glycaemia during the day or night to this elevation is not known. Our limited data do not suggest the average glucose is higher during specific shift periods in people with T2D; however, two measures of glycaemic variability during night shifts were observed to be higher than during a RAN shift: MAG and CONGA. CONGA measures short‐term variability and reflects the quality and quantity of food eaten and supports the higher energy intake observed when a night shift is worked. MAG is a measure of glucose ‘distance travelled’ over time so a higher value, even over a short time reflects variability independent of the absolute glucose value. Importantly MAG has been shown to be a good discriminator between and within individuals,^33^ and is one of the only GV metrics associated with a hard clinical outcome.^34^ Replication of this observation would inform potential importance for further investigation. Findings from the Shift‐Diabetes qualitative study found that shift workers living with T2D were concerned about the impact of poor diet and shift work on their blood glucose and perceived the provision of CGMs as of potential benefit to diabetes management.^16^ Given the complexity of interpretation of glucose variables from a research perspective, further research is needed to understand the potential benefits.^35^

During all work shifts, participants recorded a high step count, above the daily median reported in UK adults 6222.^36^ More than half of the step count was recorded during working hours. The health benefits of occupational physical activity are complex, with a suggestion of a ‘physical activity health paradox’ where occupational physical activity may not be as beneficial as leisure time physical activity^37^ and warrants further investigation in occupations with high night work activity. This also underscores the need for targeted support for night shift workers. Common guidance to increase moderate physical activity in individuals already doing a higher than average quantity of moderate physical activity is unlikely to be helpful. By contrast, strategies to encourage short bouts of more vigorous physical activity may not only be more physiologically effective,^38^ may also be welcomed as more suited to the individual circumstances.

### 5.1 Strengths and limitations

A strength of the study is the duration of data collection as previous prospective diet studies in shift workers have had shorter data collection periods or used 24‐h recalls.^39^ Therefore, this study has been able to capture diet and glucose data across transition periods of different shifts. As the Shift‐Diabetes study is a mixed methods study, the observations from the monitoring study can be integrated with the qualitative findings to gain a more complete understanding of diet behaviours and T2D management in shift workers and can be taken forward to inform intervention development.^16^, ^34^ One of the limitations of this study is that we were only able to recruit approximately half of our initial sample size. This study was severely impacted by the COVID‐19 pandemic through (i) restrictions on face‐to‐face research in higher education establishments, (ii) restrictions on advertising to the healthcare workforce due to infection control on physical posters, priority messaging of health and safety, (iii) high infection rate in the target population, and (iv) shortage of staffing making time available to participate limited or impacting on short notice rota changes. Additionally, periods of strike action across the NHS December 2022 to end of the recruitment period (March 2023) further hampered recruitment. Nevertheless, this is the first study of its kind and is able to describe glycaemic variability, diet, and movement between different behavioural days and by doing so, characterise dietary behaviours across different shifts. Further research is needed to understand the contribution that diet modification during night work can have on blood glucose profiles in employees with T2D. A further limitation that is inherent in this population is the non‐standard shift schedules and short notice rota changes during the monitoring period; however, this highlights an important challenge of conducting research in this population group. Although we asked participants to report diabetes medication usage during the study period, we did not check compliance; this may impact the glucose findings and would be of interest to capture in future studies.^40^ Self‐reported diet intake is an established limitation in nutritional research. To improve compliance, detailed instructions were given at the start of the study, along with portion size photos that have been shown to improve size estimation.^18^ The completed records were checked by the Research Assistant upon return. While statistical methods exist to estimate the potential of misreporting based on energy (kcal) intake, these have not been applied to shift workers with atypical eating patterns. It is also a possibility that participants changed their usual diet through the process of recording what they eat and drink. There is no way to determine the generalisability of our study population to the shift‐working healthcare workforce living with T2D; however, we acknowledge that the population sample is not representative of the general UK population living with T2D or NHS workforce.

### 5.2 Implications for policy and practice

The Shift‐Diabetes study highlights the irregularity of behavioural schedules and the impact on dietary intakes of an irregular shift work schedule. The impact of night work on dietary intake is unlikely to be confined to when the night shift is worked but may have a continual knock‐on effect to subsequent days. The Shift‐Diabetes study did not aim to test the impact of shift work on health outcomes; however, the findings of poor diet, extended eating windows, and inconsistent sleep timing were identified in the *Management of Hyperglycaemia in Type 2 Diabetes 2022 Consensus Report* ^41^ as associated with sub‐optimal glucose control. While longer term studies are needed to determine if shift work accelerates T2D progression, clinicians and healthcare professionals should consider if additional clinical considerations should be given to irregular shift workers living with T2D. Key observations were the variability of a ‘behavioural’ day length—this indicates that applying a standard 24‐day may not be appropriate when studying shift workers with irregular schedules, and the relatively high levels of moderate physical activity in night shift workers.

These findings taken together with the results from the qualitative Shift‐Diabetes findings highlight for guidance on management which is suited to the particular challenges and experiences of night shift workers. Employers have a duty of care to protect the health of employees. Dietary intake is modifiable and frequently viewed as an individual responsibility; however, the workplace, especially the food environment, can directly impact dietary choices. With the current UK Government moving towards pre‐NHS disease prevention, the workplace will be an important target for improving diet quality and will need to include all workers regardless of the hours worked.

## 6 CONCLUSIONS

The Shift‐Diabetes study has highlighted the impact of an irregular shift schedule on dietary behaviours. Further research is needed to understand the long‐term impacts of irregular shift work in employees living with T2D. However, based on the current evidence regarding T2D management, an immediate priority should be the development of tailored interventions to support night shift‐workers with T2D to manage their condition.

## AUTHOR CONTRIBUTIONS

RG, NG, FL, NO, and BM derived the research question and designed the study. MD collected the data. MD, RG, FT derived the diet, glucose, and activity variables. LP and RG designed the statistical analysis plan. CV reviewed and commented on the analytical plan. RG and LP conducted the analyses. Manuscript was drafted by MD, NG, and RG. All authors contributed to data interpretation and writing of the final manuscript.

## FUNDING INFORMATION

The Shift‐Diabetes study is supported by Diabetes UK (grant number 19/0005979). Discounted CGM supplies were provided by Dexcom, Inc. (OUS‐2019‐046). Neither organization had any role in the study design, management, data analysis and interpretation, writing of the report, or the decision to submit the report for publication.

## CONFLICT OF INTEREST STATEMENT

RG is a volunteer member of the British Dietetic Association Work Ready Steering Group.

## Supporting information


Data S1.

## Data Availability

The data that support the findings of this study are available from the corresponding author upon reasonable request.
