# Supplementary material for: Characterising the impact of shift work on diet and glucose variability in healthcare employees living with type 2 diabetes: The Shift‐Diabetes study
Source: Diabet Med. 2026 Feb 24;43(5):e70262. doi: 10.1111/dme.70262 (PMC13074139; doi:10.1111/dme.70262)
Supplement: Supplementary file 1 — Data S1. [file DME-43-e70262-s001.docx]

**Characterising the impact of shift work on diet and glucose variability in healthcare employees living with type 2 diabetes: The Shift-Diabetes Study**

**Supplementary tables and figures**

| Date | Monitoring day number | Activity | Time | Duration calculation – sleep off set to sleep off set |
| --- | --- | --- | --- | --- |
| 2nd Feb | day 1 | wake up | 05:10 | day off day duration: 24hrs 20 minutes |
|  | day 1 | shift time | day off |  |
|  | day 1 | start sleep | 21:47 |  |
| 3rd Feb | day 1 / 2 | wake up | 05:30 | day shift day duration: 24 hours |
|  | day 2 | shift time | 8am to 8:30pm |  |
|  | day 2 | start sleep | 22:45 |  |
| 4th Feb | day 2 / 3 | wake up | 05:30 | day shift day duration: 29 hrs 20 mins |
|  | day 3 | shift time | 8am to 8:30pm |  |
|  | day 3 | start sleep | 23:10 |  |
| 5th Feb | day 3/4 | wake up | 10:40 | day off day duration: 19hrs 32 mins |
|  | day 4 | shift time | day off |  |
|  | day 4 | start sleep | 11:05 |  |
| 6th Feb | day 4/5 | wake up | 06:12 | day off duration: 24 hrs 33 mins |
|  | day 5 | shift time | day off |  |
|  | day 5 | start sleep | 21:51 |  |
| 7th Feb | day 5/6 | wake up | 07:45 | night shift duration: 32 hrs 40mins |
|  | day 6 | shift time | 20:00 to 08:30 (8^th^ Feb) |  |
| 8^th^ Feb | day 6 | start sleep | 10:20 |  |
| 8th Feb | day 6/7 | wake up | 16:20 |  |
|  | day 7 | shift time | 20:00 to 08:30 (9^th^ Feb) |  |
| 9^th^ Feb | day 7 | start sleep | 10:40 |  |

**Figure 1** Worked example of behavioural day duration calculation and classification from Shift Diabetes participant

Assessed for eligibility: n = 84

Excluded: n=37

Not meeting inclusion criteria (n=27)

- Not working nights/enough nights (n=10)
- Started a new medication (n=1)
- Not T2D diagnosis (n=1)
- Permanent night workers (n=4)
- Taking insulin or sulphonylureas (n=6)
- Not working hospital/residential care environment (n=2)
- Medical history (n=3)

Lost contact (n=10)

## Enrolment

Consented: n = 47

- Lost contact (n=7)

## Monitoring study

Started monitoring study: n = 40

- Did not complete study (n = 1)
- Actual shift schedule did not meet protocol requirements (n = 1)
- Incomplete reporting (n = 1)

Monitoring study completed: n = 37

Missing CGM data = 1

(n = 36 included for CGM analyses)

Missing activity monitoring data = 2

Incomplete across all shift types = 3

(n= 34 for complete analyses)

## Analysis

**Figure S2.** CONSORT flow diagram Shift-Diabetes

**Table S1**: Summary of total diet intake for Shift Diabetes participants across the total monitoring period (*n* = 37)

|  | Mean | (SD) |
| --- | --- | --- |
| Tot energy kcal | 1936.7 | 539.5 |
| Carbohydrates (% EI) | 42.7 | 7.4 |
| Tot fats (% EI) | 38.5 | 6.9 |
| Saturated fats (% EI) | 13.2 | 3.1 |
| Protein (% EI) | 17.8 | 3.1 |
| Free sugars g-day | 39.6 | 21.2 |
| Salt g/day | 6.1 | 2.5 |
| Fibre g/day (AOAC) | 20.1 | 10.3 |
| Fruit g/day | 98.8 | 78.3 |
| Vegetables g/day | 222.2 | 586.5 |
| Wholegrains g/day | 42.3 | 57.4 |
| SSB kcal/day | 16.05 | 26.8 |
| Sweet snacks kcal/day | 236.3 | 188.3 |
| Alcohol g/day (ethanol) | 3.1 | 5.3 |
| Caffeine mg/day | 113 | 109.6 |

**Table S2**: Proportion of Shift-Diabetes participants meeting key UK healthy eating recommendations (1) (n=37)

|  | n | % |
| --- | --- | --- |
| Saturated fat (men <30g/day, women <20g/day) | 9 | 24.3 |
| Fibre >30g/day | 4 | 10.8 |
| Fruit and vegetables >400g/day | 2 | 5.4 |
| Salt <6g/day | 22 | 60 |
| Free sugar <30g/day | 10 | 27 |

1. Public Health England, 2016 [online] https://assets.publishing.service.gov.uk/media/5a749fece5274a44083b82d8/government_dietary_recommendations.pdf

**Table S3** Summary of continuous glucose and wear time variables for Shift-Diabetes participants (n = 36)

|  | Mean (SD) | |
| --- | --- | --- |
| Mean readings per day, n | 287 | 3.6 |
| Mean %of CGM data capture across 10 days | 94 | 16.5 |
| Mean HbA1c mmol/mol | 57.0 | 14.8 |
| Mean blood glucose (MBG) mmol/L | 9.14 | 2.14 |
| SD, mmol/L | 1.93 | 0.54 |
| Coefficient of variation (CV), % | 20.9 | 3.8 |
| Mean absolute glucose (MAG), mmol/L | 2.55 | 0.55 |
| Mean amplitude of glycaemic excursion (MAGE), mmol/L | 4.63 | 1.45 |
| Continuous overlapping net glycaemic action (CONGA), mmol/L | 2.22 | 0.58 |
| Mean of daily differences (MODD), mmol/L | 1.88 | 0.56 |
| Time in range (3.9 – 10.00mmol/L), % | 68.02 | 27.05 |
| Time in severe (>13.9mmol/L), % | 4.41 | 11.25 |

**Table S4** Self-reported sleep parameters of Shift Diabetes participants (n = 37)

| Summary outcome^1^ | n | % |
| --- | --- | --- |
| *Sleep related impairment* |  |  |
| None to slight | 23 | 62.2 |
| Mild | 6 | 16.2 |
| Moderate | 5 | 13.5 |
| Severe | 1 | 2.7 |
| *Sleep disturbance* |  |  |
| None to slight | 21 | 56.8 |
| Mild | 8 | 21.6 |
| Moderate | 5 | 13.5 |
| Severe | 2 | 5.4 |
| *Sleep Apnoea risk category^2^* |  |  |
| High | 20 | 54.1 |
| Low | 17 | 45.9 |
| *Chronotype^3^* |  |  |
| Definitely a morning type | 8 | 21.6 |
| Rather more morning than evening | 4 | 10.8 |
| Don’t know | 2 | 5.4 |
| Rather more evening than morning | 9 | 24.3 |
| Definitely an evening type | 14 | 37.8 |
| *Lightness of room where you sleep (%)* |  |  |
| Light enough to read | 5 | 13.5 |
| Light enough to see across the room but not to read | 16 | 43.2 |
| Light enough to see your hand in front of you, but not across the room | 7 | 18.9 |
| Too dark to see your hand or wear an eye mask | 9 | 24.3 |

1 PROMIS (post monitoring period), missing data from questionnaire n = 2 SRI, n = 1 SD; 2 Berlin Questionnaire (collected at baseline) n= 39. 3.Chronotype one question self-report
